# Unraveling and engineering the production of 23,24-bisnorcholenic steroids in sterol metabolism

**DOI:** 10.1038/srep21928

**Published:** 2016-02-22

**Authors:** Li-Qin Xu, Yong-Jun Liu, Kang Yao, Hao-Hao Liu, Xin-Yi Tao, Feng-Qing Wang, Dong-Zhi Wei

**Affiliations:** 1 State Key Laboratory of Bioreactor Engineering, Newworld Institute of Biotechnology, East China University of Science and Technology, Shanghai 200237, China

## Abstract

The catabolism of sterols in mycobacteria is highly important due to its close relevance in the pathogenesis of pathogenic strains and the biotechnological applications of nonpathogenic strains for steroid synthesis. However, some key metabolic steps remain unknown. In this study, the *hsd4A* gene from *Mycobacterium neoaurum* ATCC 25795 was investigated. The encoded protein, Hsd4A, was characterized as a dual-function enzyme, with both 17β-hydroxysteroid dehydrogenase and β-hydroxyacyl-CoA dehydrogenase activities *in vitro*. Using a *kshAs-*null strain of *M. neoaurum* ATCC 25795 (NwIB-XII) as a model, Hsd4A was further confirmed to exert dual-function in sterol catabolism *in vivo*. The deletion of *hsd4A* in NwIB-XII resulted in the production of 23,24-bisnorcholenic steroids (HBCs), indicating that *hsd4A* plays a key role in sterol side-chain degradation. Therefore, two competing pathways, the AD and HBC pathways, were proposed for the side-chain degradation. The proposed HBC pathway has great value in illustrating the production mechanism of HBCs in sterol catabolism and in developing HBCs producing strains for industrial application via metabolic engineering. Through the combined modification of *hsd4A* and other genes, three HBCs producing strains were constructed that resulted in promising productivities of 0.127, 0.109 and 0.074 g/l/h, respectively.

Utilizing natural sterols, such as cholesterol and phytosterols, as carbon and energy sources is a common physiological attribute of certain actinomycetes, such as mycobacteria and rhodococci^1^,^2^,^3^,^4^,^5^,^6^,^7^. The catabolism of sterols in pathogenic strains, such as *M. tuberculosis*, is highly concerned due to its close relevance to pathogenesis and persistence^1^,^2^. Interestingly, sterol metabolism in nonpathogenic microorganisms generates metabolites that can be used as ideal precursors to synthesize steroidal pharmaceuticals (Fig. 1)^8^,^9^. Generally, two major valuable intermediates can be achieved from the metabolism of sterols, including C19 steroids and C22 steroids (23,24-bisnorcholenic steroids, HBCs), of which the main difference is the side chain located at carbon 17 (Fig. 1)^8^,^10^,^11^. In industrial manufacturing, C19 steroids, including androst-4-ene-3,17-dione (AD), androst-1,4-dien-3,17-dione (ADD) and 9α-hydroxy-androst-4-ene-3,17-dione (9-OHAD), can be used to synthesize sex and adrenocortical hormones, which have been massively produced due to the successful development of industrial strains by metabolic engineering or mutation breeding (Fig. 1)^4^,^10^. However, the production of C22 steroids is less optimal, as ideal industrial strains have yet to be developed, although some C22 steroids, including 22-hydroxy-23,24-bisnorchol-4-ene-3-one (4-HBC), 22-hydroxy-23,24-bisnorchol-1,4-dien-3-one (1,4-HBC) and 9,22-dihydroxy-23,24-bisnorchol-4-ene-3-one (9-OHHBC), are highly valuable precursors in synthesizing progestational and adrenocortical hormones (Fig. 1)^8^,^9^.

The metabolism of sterols in actinomycetes follows a similar pathway, which is initiated by the conversion of sterols to 4-ene-3-sterone, catalyzed by cholesterol oxidases or 3β-hydroxy steroid dehydrogenases^3^,^12^,^13^. The catabolic pathway then can be divided into two parts: the cleavage of the steroid skeleton and the degradation of the side chain^1^,^14^,^15^. The opening of the steroid skeleton has been well studied and is performed by the combined 1,2-desaturation and 9-hydroxylation catalyzed by 3-ketosteroid-Δ^1^-dehydrogenases (KstDs) and 3-ketosteroid-9α-hydroxylases (KSHs), respectively^16^,^17^. The blockage of 1,2-desaturation or/and 9-hydroxylation by genetic manipulation or mutagenesis blocks complete sterol catabolism and results in the production of C19 steroids^4^,^18^. However, the side-chain degradation of sterols is quite complicated and has yet to be completely understood^1^,^19^. The sterol side-chain degradation is proposed to proceed in a manner similar to the β-oxidation of fatty acids, which is initiated by a terminal carboxylation by Cyp125^20^,^21^. The side-chain degradation then is proposed to comprise three β-oxidation-like cycles, which each cycle containing successive enzymatic steps catalyzed by acyl-CoA ligases, acyl-CoA dehydrogenases, enoyl-CoA hydratases, β-hydroxyacyl-CoA-dehydrogenases and acyl-CoA thiolases^1^,^18^. To date, several steps in the β-oxidation-like cycles have been characterized, such as the thiolysis of 3,22-dioxo-chol-4-ene-24-oyl-CoA to 3-OPC-CoA catalyzed by FadA5 and the dehydrogenation of 3-oxo-4-pregnene-20-carboxyl-CoA catalyzed by FadE28-FadE29^2^,^22^. However, some key steps involved in the sterol side-chain degradation remain unknown, restricting a complete understanding of this process.

*Hsd4A* is a putative gene located in the sterol catabolic gene cluster, which was deduced to encode a short chain dehydrogenase (Hsd4A). As Hsd4A shares intriguing similarity with the N-terminal domain of eukaryotic 17β-hydroxysteroid dehydrogenase IV (17βHSD4), Hsd4A has been speculated to be a 17β-hydroxysteroid dehydrogenase^15^,^23^. However, the *hsd4A* gene in *R. jostii* RHA1 was highly up-regulated along with other genes that are involved in the side-chain degradation of sterols, and thus was speculated to be essential for sterol side-chain degradation^15^. Therefore, Hsd4A was further proposed to be a β-hydroxyacyl-CoA dehydrogenase in the degradation of the sterol side chain because the N-terminal domain of 17βHSD4 also could act as a D-3-hydroxyacyl-CoA dehydrogenase in degrading branched fatty acids and bile acids^15^,^23^,^24^. Although these two possible biochemical functions have been assigned to Hsd4A, neither have been validated^1^. As these two putative functions may have underlying associations in the production of C19 or C22 steroids, the exact function of Hsd4A requires elucidation.

With the characterization of *kstD*s and *kshA*s homologous genes in some actinomycetes^17^,^25^,^26^,^27^,^28^, it has become feasible to develop C19 steroids producing strains with ideal productive performance by metabolic engineering^4^,^29^. However, C22 steroids producing strains cannot be developed by rational genetic engineering due to the unknown formation mechanism of C22 steroids. Early studies identified C22 steroids as the byproducts of the transformation of sterols to C19 steroids in several *Mycobacterial* strains acquired by mutagenesis, such as *Mycobacterium sp.* NRRL B-3683 and *M. fortuitum* NRRL B-8119^30^,^31^,^32^. For example, only about 55 μmol bisnorcholenic acid derivatives and 9-OHHBC can be produced from transforming β-sitosterols to 700 μmol of 9-OHAD by *M. fortuitum* NRRL B-8119^32^,^33^. Obviously, these C19 steroids producing mycobacteria are not the desired strains to produce C22 steroids. To develop attractive C22 steroids producing strains, mutation breeding method has been utilized. For example, *M. parafortuitum* complex MCI 0617 was developed using ultraviolet mutagenesis, and this strain was able to produce 5.3 g/l of 1,4-HBC, along with two byproducts 4-HBC (about 0.44 g/l) and ADD (0.06 g/l) from 10 g/l of cholesterol^11^. However, the development of C22 steroids producing strains has been largely unsuccessful because of the low productivity and the presence of multiple byproducts, which greatly limits the commercial application of these strains.

While investigating the role of *hsd4A* in sterol metabolism, we discovered that *hsd4A* might be a key gene relating to the formation of C22 steroids. In this study, therefore, we attempted to investigate the physiological role of Hsd4A and its possible mechanism in the conversion of sterols to C22 steroids, based on which we also tried to establish a rational strategy to modify *hsd4A* and other key genes to develop attractive HBCs producing strains.

## Results

### *Hsd4A*_*MN*_ gene from *M. neoaurum* ATCC 25795

The sterol catabolic gene cluster in *M. neoaurum* ATCC 25795 is described schematically in Fig. 2a. In the gene cluster, a putative gene *hsd4A* (*hsd4A*_*MN*_) was annotated as an ortholog of the *hsd4A* gene (Rv3502c) from *M. tuberculosis* H37Rv. Its encoded protein, Hsd4A_MN_, was estimated to be 30.9 kDa and shares high similarities with its counterparts, such as 97%, 71% and 67% amino acid identities with the Hsd4As from *Mycobacterium* sp.VKM Ac-1815D, *M. tuberculosis* H37Rv and *R*. *jostii* RHA1, respectively (Fig. 2b). As shown in Fig. 2, *hsd4A*_*MN*_ is located between the *mce4* operon and *fadE26*–*27*. The *mce4* operon encodes a multicomponent ABC-like ATP-dependent transport system, which plays an important role in the cellular uptake of steroids^1^,^15^,^34^. The *fadE26*–*27* locus encodes acyl-CoA dehydrogenase enzymes that catalyze the dehydrogenation of 3-oxo-cholest-4-en-26-oyl CoA in the first cycle of cholesterol side-chain β-oxidation^35^. Obviously, *hsd4A*_*MN*_ and its genetic organization are highly conserved in some mycobacteria and rhodococci (Fig. 2b), indicating the conserved function of Hsd4A in these strains for sterol catabolism. Hsd4As from *M. tuberculosis* H37Rv and *R. jostii* RHA1 have been proposed to be a β-hydroxyacyl-CoA dehydrogenase or a 17β-hydroxysteroid dehydrogenase^1^,^15^,^23^,^36^, indicating a possibly similar function for Hsd4A_MN_. However, the exact role of Hsd4A in these microorganisms has not been characterized^1^.

### Enzymatic characteristics of Hsd4A_MN_ *in vitro*

To explore the possible function, *hsd4A*_*MN*_ was expressed heterologously in *Escherichia coli* BL21(DE3) (E-*hsd4A*, Table S1). The proposed activities of Hsd4A_MN_ were then investigated using purified protein (Fig. S1). As indicated in Table 1, Hsd4A_MN_ catalyzed the dehydrogenation of testosterone (TS) and boldenone (BD) in the presence of NAD^+^ to produce AD and ADD, respectively. Interestingly, it did not catalyze the 17β-reduction of AD and ADD in the presence of NADH, demonstrating that Hsd4A_MN_ does have 17β-hydroxysteroid dehydrogenase function without reversible activity. As for the proposed β-hydroxyacyl-CoA dehydrogenase activity of Hsd4A_MN_, two commercially available fatty acyl-CoA esters, acetoacetyl-CoA and DL-β-hydroxybutyryl-CoA, were tested as substrates for Hsd4A_MN_. The results showed that Hsd4A_MN_ catalyzed the conversion of β-hydroxybutyryl-CoA to acetoacetyl-CoA using NAD^+^ as coenzyme and also carried out the reverse reaction in the presence of NADH, which unambiguously demonstrated that Hsd4A_MN_ also had β-hydroxyacyl-CoA dehydrogenase function with reversible catalytic activity (Table 1). According to the above results, therefore, Hsd4A_MN_ was characterized as a dual-function enzyme *in vitro*, possessing both the activities of β-hydroxyacyl-CoA dehydrogenase and 17β-hydroxysteroid dehydrogenase.

To further characterize Hsd4A_MN_, its enzymatic properties were determined (Table 1). Compared to TS and BD, 4-androstene-17α-ol-3-one (epi-TS) and 4-androstene-17α-methyl-17β-ol-3-one (17α-MT) could not be catalyzed by Hsd4A_MN_, confirming that Hsd4A_MN_ has the typical activity of 17β-hydroxysteroid dehydrogenase with high regio and stereoselectivity. Compared to acetoacetyl-CoA and DL-β-hydroxybutyryl-CoA, acetoacetate and DL-β-hydroxybutyric acid could not be converted by Hsd4A_MN_, indicating that the enzyme is a typical β-hydroxyacyl-CoA dehydrogenase similar to the counterparts involved in fatty acid β-oxidation. The kinetic parameters *K*_m_ and *V*_max_ of Hsd4A_MN_ for TS and BD were 19–23 μM and 0.169–0.217 μmol/min/mg, respectively; and for acetoacetyl-CoA and DL-β-hydroxybutyryl-CoA were 101–124 μM and 0.091–0.113 μmol/min/mg, respectively.

### Physiological role of Hsd4A_MN_ *in vivo*

Hsd4A_MN_ acts as a dual-function enzyme *in vitro*, but its physiological role *in vivo* is unknown. As *hsd4A*_*MN*_ is highly conserved in the sterol catabolic gene cluster, the investigation mainly focused on its role in sterol metabolism. Corresponding to the enzymatic activities *in vitro*, Hsd4A_MN_ were deduced to have two roles in sterol metabolism *in vivo*: the oxidation of 17β-hydroxysteroids by 17β-hydroxysteroid dehydrogenase and the β-hydroxyacyl-CoA dehydrogenation in the β-oxidation of the sterol side chain by β-hydroxyacyl-CoA dehydrogenase. Both of these reactions can occur before the cleavage of the steroid nucleus in sterol metabolism. Given that the natural sterols can be completely degraded by *M. neoaurum* ATCC 25795 to CO_2_ and H_2_O without obvious accumulation of intermediates (Fig. 3a), the function of Hsd4A_MN_ is difficult to determine via identifying the metabolites of sterols after genetic manipulation of *hsd4A*_*MN*_ in *M. neoaurum* ATCC 25795. To facilitate the functional analysis of Hsd4A_MN_, therefore, it was necessary to build a model strain which could accumulate suitable metabolites with intact steroid skeletons before the genetic manipulation of *hsd4A*_*MN*_.

3-Ketoseroid-9α-hydroxylase (encoded by two genes, *kshA* and *kshB*) is one of the key enzymes in dissociation of the steroid skeleton, and inactivating this enzyme would block the cleavage of the steroid skeleton^4^,^28^. Based on the genome sequence, two putative *kshA* (*kshA1* and *kshA2*) genes were identified in *M. neoaurum* ATCC 25795 (Fig. 2a). Therefore, a model to investigate the function of Hsd4A_MN_ *in vivo* was constructed by the successive deletion of *kshA1* and *kshA2* in *M. neoaurum* ATCC 25795, which is a *kshAs*-null mutant (NwIB-XII, Table S1). As expected, sterol catabolism in strain NwIB-XII was blocked, accumulating multiple metabolites with intact steroid skeletons, including ADD (I), AD (II), and two 17β-hydroxysteroids TS (III) and BD (IV) (Figs 3a,b and S2).

To determine whether Hsd4A_MN_ possesses 17β-hydroxysteroid dehydrogenase activity *in vivo*, *hsd4A*_*MN*_ was augmented in NwIB-XII using plasmid pMV261-*hsd4A*, resulting in the XIIp261*hsd4A* strain. The vacant plasmid pMV261 was introduced into NwIB-XII as a control (designated as XIIp261). In contrast to NwIB-XII and XIIp261, strain XIIp261*hsd4A* transformed sterols to AD and ADD without any detected TS and BD (Figs 3a and S3), which could be ascribed to the conversion of TS and BD to AD and ADD, respectively, by the enhanced Hsd4A_MN_ activity (Fig. 3b). Therefore, this suggests that Hsd4A_MN_ can also act as a 17β-hydroxysteroid dehydrogenase to irreversibly catalyze the oxidation of TS to AD and BD to ADD *in vivo*.

To further characterize the function of Hsd4A_MN_, *hsd4A*_*MN*_ was deleted in NwIB-XII, leading to the XIIΔ*hsd4A* strain (Table S1). Surprisingly, the metabolic products of sterols in XIIΔ*hsd4A* were significantly different from those in NwIB-XII. XIIΔ*hsd4A* converted sterols to two novel products, V and VI (Fig. 4a). The major product V was identified as 1,4-HBC and the minor product VI was identified as 4-HBC (Figs 4b and S4). Both 1,4-HBC and 4-HBC are the incompletely degraded products of the sterol side chain, which still contain three carbon atoms at carbon 17. To confirm whether the production of these two compounds could be attributed to the deletion of *hsd4A*_*MN*_, *hsd4A*_*MN*_ was then complemented in XIIΔ*hsd4A* (strain Cp-4A, Table S1), which restored the same metabolic phenotype observed in NwIB-XII (Fig. S5). These results confirmed that deletion of *hsd4A*_*MN*_ resulted in incomplete degradation of the sterol side chain, demonstrating that Hsd4A_MN_ plays a significant role in sterol side-chain degradation. Therefore, Hsd4A_MN_ exhibited two enzymatic activities *in vivo*, simialr to results observed *in vitro*. According to the characterized enzymatic properties of Hsd4A_MN_ *in vitro*, the involvement of Hsd4A_MN_ in side-chain degradation should be attributed to its β-hydroxyacyl-CoA dehydrogenase activity, which has been proposed in previous studies^1^,^15^,^36^.

Although 1,4-HBC and 4-HBC were the products derived from the deletion of *hsd4A*_*MN*_, they were verified to be not the substrates of Hsd4A_MN_ *in vitro* (Table 1), indicating that the two compounds are further derivatives of the physiological substrate of Hsd4A_MN_. According to the postulated β-oxidation of the sterol side chain, the most probable precursor of 1,4-HBC and 4-HBC is 22-hydroxy-3-oxo-25,26-bisnorchol-4-en-24-oyl CoA (22HOBNC-CoA), which contains a β-hydroxybutyryl-CoA moiety that has been determined to be the active substrate of Hsd4A_MN_ *in vitro* (Table 1).

### Engineering of the metabolism of sterols to produce HBCs

**Figure 1:**
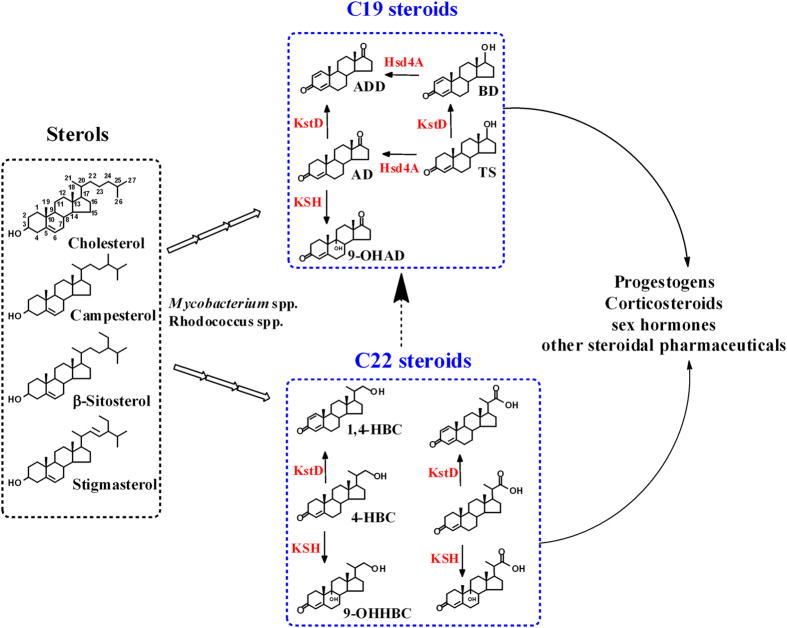
An overview of the biotransformation of sterols to steroidal pharmaceuticals. Sterols, including cholesterol and phytosterols, can be degraded to two major valuable intermediates, such as C19 and C22 steroids, through multiple biotransformation steps by some actinomyces, such as *Mycobacterium* spp. and *Rhodococcus* spp. Well-studied C19 steroids are AD (androst-4-ene-3,17-dione), ADD (androst-1,4-dien-3,17-dione), 9-OHAD (9α-hydroxy-androst-4-ene-3,17-dione), BD (boldenone) and TS (testosterone). Common C22 steroids are 4-HBC (22-hydroxy-23,24-bisnorchol-4-ene-3-one), 1,4-HBC (22-hydroxy-23,24-bisnorchol-1,4-dien-3-one) and 9-OHHBC (9,22-dihydroxy-23,24-bisnorchol-4-ene-3-one). C22 steroids can be degraded to C19 steroids through multiple undefined biotransformation steps. These two intermediates can be used as ideal precursors to synthesize steroidal pharmaceuticals, such as progestogens and corticosteroids. The abbreviations in red represent the key enzymes that determine product selectivity. KstD, 3-ketosteroid-Δ^1^-dehydrogenases; KSH, 3-ketosteroid-9α-hydroxylases; Hsd4A, a short chain dehydrogenase. Arrows in arrays signify multiple biotransformation steps. Curved arrows indicate the application value of C22 steroids and C19 steroids to produce steroidal pharmaceuticals. The dashed arrow indicates undefined biotransformation steps.

**Figure 2:**
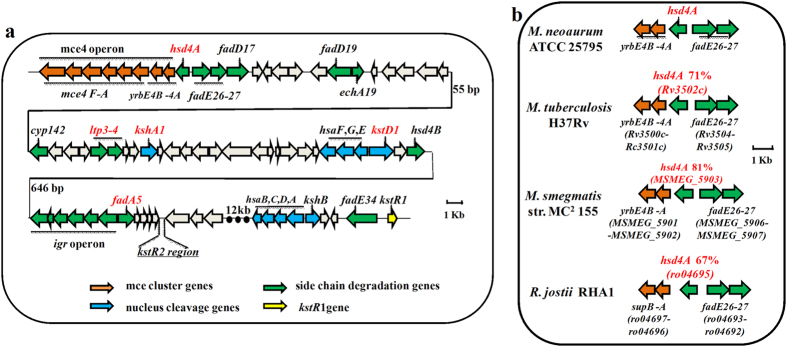
(**a**) Partial gene cluster encoding the catabolism of sterols in *M. neoaurum* ATCC 25795. Genes in the map are color-coded according to assigned or proposed function: orange, mce cluster genes for steroids transportation; green, side-chain degradation genes; blue, nucleus cleavage genes; gray, unassigned function; yellow, gene encoding the transcriptional repressor KstR1. The numbers, e.g. 55 bp and 646 bp, beside the meander lines indicate the spaces between adjacent genes. (**b**) Schematic of the genomic organization of *hsd4A* homologues in *M. neoaurum* ATCC 25795 and other mycobacteria. Percentages, such as 71%, 81% and 67%, indicate the amino acid sequence identity of Hsd4A_MN_ in *M. neoaurum* ATCC 25795 with homologs from other mycobacteria, including *M. tuberculosis* H37Rv, *M. smegmatis* str. MC^2^ 155 and *R*. *jostii* RHA1. The location of *hsd4A* is between the *yrbE4B-4A* and *fadE26*–*27*. The size and direction of genes are indicated by arrows with corresponding length to scale.

In the industry, HBCs (including 1,4-HBC, 4-HBC and 9-OHHBC, Fig. 1) can be used as precursors to produce progestational and adrenocortical hormones. HBCs are mainly produced from the modified metabolism of sterols in microorganisms. However, the formation mechanism of HBCs in sterol metabolism has not been clarified, and industrial strains used to produce HBCs are usually achieved by mutation breeding^11^. Therefore, it is a great challenge to rationally develop HBCs producing strains. In this study, the production of HBCs in the *hsd4A*_*MN*_- deleted strain opened the door to unravel the production mechanism of HBCs and paved the way to rationally construct HBCs producing strains.

As indicated in Fig. 4a, strain XIIΔ*hsd4A* could transform sterols to HBCs. To evaluate the production performance, a resting cell biotransformation of physterols was employed. When 40 g/l of phytosterols was fermented by XIIΔ*hsd4A* resting cells for 144 h, a 39–40% molar yield (12.48–13.04 g/l) of 1,4-HBC was achieved, along with an 18–20% yield (6.02–6.47 g/l) of 4-HBC and a 1–2% yield (0.41–0.53 g/l) of ADD (Table 2). This performance made the strain XIIΔ*hsd4A* not ideal for industrial application because the low yield ratio of 4-HBC or 1,4-HBC among the products would lead to a low recovery rate at the industrial scale. Therefore, a combined strategy to further modify the metabolic pathway of sterols was necessary to develop promising strains for industrial application.

1,4-HBC and 4-HBC differ at the C1, 2 double bond, which is catalyzed by KstDs^4^,^6^,^25^. There are three KstD homologues in *M. neoaurum* ATCC 25795, and KstD1 was characterized as the major enzyme in our previous work^4^. Therefore, strain XIIΔ*hsd4A* could be further optimized to better produce 1,4-HBC or 4-HBC by strengthening or weakening the activities of KstDs (Fig. 5a,b).

To develop the 1,4-HBC producing strain, *kstD1* was overexpressed in XIIΔ*hsd4A* (pMV261-*kstD1*), resulting in the XIIΔ*hsd4A*-p261*kstD1* strain (Fig. 5a). The transformation of 40 g/l of phytosterols by resting cells for 144 h resulted in a 54–57% molar yield of 1,4-HBC (17.56–18.23 g/l) with a 4–7% yield of 4-HBC (1.57–2.15 g/l) and a 1–2% yield (0.35–0.52 g/l) of ADD (Table 2). The overexpression of *kstD1* in XIIΔ*hsd4A* significantly increased the yield ratio of 1,4-HBC among the products from 65–66% to about 90% and the productivity of 1,4-HBC from 0.087–0.091 g/l/h to 0.122–0.127 g/l/h, greatly improving the industrial applicability for 1,4-HBC production.

To construct the 4-HBC producing strain, *kstD1* was deleted in XIIΔ*hsd4A*, resulting in a strain XIIΔ*hsd4A*Δ*kstD1* (Fig. 5b). The deletion of *kstD1* increased the production of 4-HBC from an 18–20% molar yield (6.02–6.47 g/l) to a 33–35% molar yield (10.68–11.22 g/l) after the transformation of 40 g/l of phytosterols. However, there was still a 15–18% molar yield of 1,4-HBC (5.02–5.77 g/l) (Table 2). In our previous study, *kstD3* was demonstrated to be another important gene encoding KstD^4^. Therefore, *kstD3* was further deleted in XIIΔ*hsd4A*Δ*kstD1* to weaken the yield of 1,4-HBC. Unexpectedly, in the transformation of 40 g/l of phytosterols for 144 h, strain XIIΔ*hsd4A*Δ*kstD13* still accumulated significant amounts of 1,4-HBC (4.35–4.86 g/l), indicating that there was significant residual KstD activity. Therefore, *kstD2*, which was characterized as a weak KstD gene^4^, was also deleted, resulting in a *kstDs*-null mutant XIIΔ*hsd4A*Δ*kstD123*. The transformation of 40 g/l phytosterols by XIIΔ*hsd4A*Δ*kstD123* achieved a 47–49% molar yield (15.24–15.75 g/l) of 4-HBC, along with a 1–2% yield (0.52–0.61 g/l) of 1,4-HBC, a 2–3% yield (0.93–1.04 g/l) of compound VII and a small amount of AD (0.31–0.42 g/l) (Table 2). Among these products, the yield ratio of 4-HBC reached about 90%, and its productivity was increased to 0.106–0.109 g/l/h, indicating that XIIΔ*hsd4A*Δ*kstD123* was a good 4-HBC producing strain (Table 2).

The compound VII was identified as 9-OHHBC (Fig. S4), which is a useful precursor to synthesize adrenocorticoids. Therefore, the feasibility in developing a 9-OHHBC producing strain was investigated. According to the steroid nucleus metabolic mechanism (Fig. 5c), *kstD1*, *kstD2*, *kstD3* and *hsd4A*_*MN*_ were progressively deleted in *M. neoaurum* ATCC 25795, resulting in the final strain MNΔ*hsd4A*Δ*kstD123*. In the transformation of 40 g/l of phytosterols, strain MNΔ*hsd4A*Δ*kstD123* produced a 30–32% molar yield of 9-OHHBC (10.25–10.72 g/l) (Table 2), demonstrating that a 9-OHHBC producing strain could be developed by combined modification of the key enzyme genes *hsd4A*_*MN*_ and *kstD*s. In terms of industrial applications, strain MNΔ*hsd4A*Δ*kstD123* should be further optimized because of the massive accumulation of the byproduct 9-OHAD.

In conclusion, *hsd4A*_*MN*_ plays a key role in the production of HBCs during sterol metabolism, and the combined modification of *hsd4A*_*MN*_ and other key genes is a useful strategy to develop HBCs producing strains. Compared to C19 steroids producing strains in the industry (typically produce 12–13 g/l of C19 steroids from 30 g/l of phytosterols after 120 h), these HBCs producing strains displayed to be promising for industrial applications.

## Discussion

**Figure 3:**
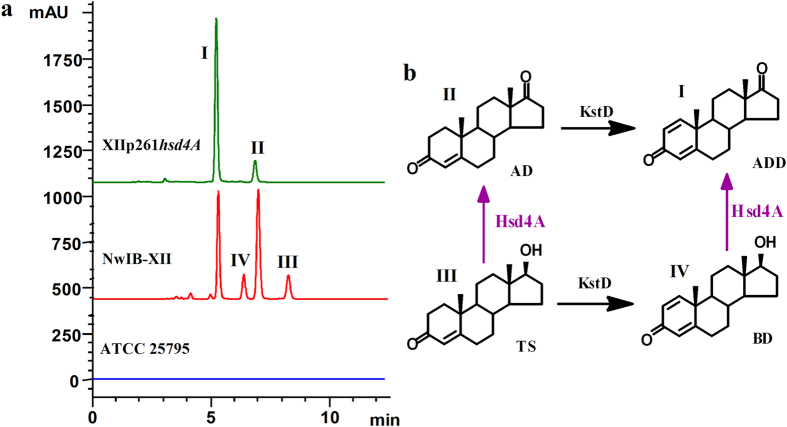
Phenotypic analyses of the metabolism of sterol by *M. neoaurum* ATCC25795 and its derivative strains. (**a**) HPLC chromatogram comparison of the products from the transformation of 2 g/l of cholesterol in MYC/02 media at 30 °C by strains *M. neoaurum* ATCC 25795 (blue), NwIB-XII (red) and XIIp261*hsd4A* (green). Cholesterol can be completely degraded by *M. neoaurum* ATCC 25795 without obvious accumulation of intermediates. The catabolism of cholesterol in NwIB-XII was blocked to accumulate multiple metabolites, including ADD (I), AD (II), TS (III) and BD (IV). The plasmid pMV261-*hsd4A* was electro-introduced into NwIB-XII, resulting in the XIIp261*hsd4A* strain. Strain XIIp261*hsd4A* transformed cholesterol to ADD (I) and AD (II). (**b**) Conversion relationship between the metabolites (I to IV). Arrows in purple signify the function of Hsd4A_MN_ deduced from the product phenotypes of strains NwIB-XII and XIIp261*hsd4A*. Hsd4A_MN_ can irreversibly catalyze the oxidation of TS to AD and BD to ADD, respectively. AD, androst-4-ene-3,17-dione; ADD, androst-1,4-dien-3,17-dione; BD, boldenone; TS, testosterone.

**Figure 4:**
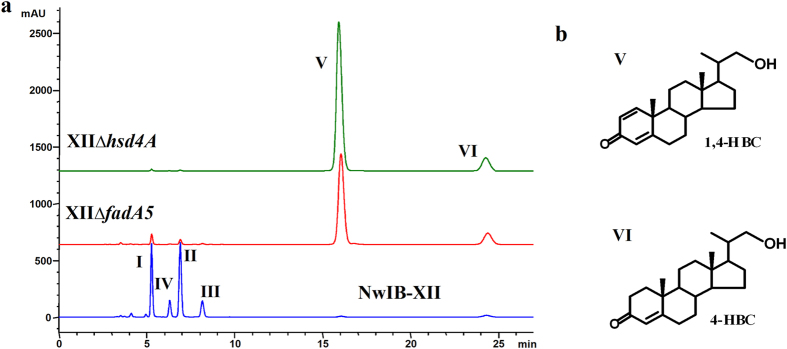
Phenotypic analyses of the sterol metabolism of *M. neoaurum* NwIB-XII and its derivative strains. (**a**) HPLC chromatogram comparison of the products from the transformation of 2 g/l of cholesterol in MYC/02 media at 30 °C by strains NwIB-XII (blue), XIIΔ*fadA5* (red) and XIIΔ*hsd4A* (green). The deletion of *hsd4A*_*MN*_ or *fadA5* in NwIB-XII greatly changed the metabolic products of cholesterol. Both XIIΔ*hsd4A* and XIIΔ*fadA5* converted cholesterol to 1,4-HBC (V) and 4-HBC (VI). (**b**) 23,24-Bisnorcholenic steroids characterized from the metabolites accumulated in *M. neoaurum hsd4A*_*MN*_or *fadA5* deleted strains. 1,4-HBC, 22-hydroxy-23,24-bisnorchol-1,4-dien-3-one; 4-HBC, 22-hydroxy-23,24-bisnorchol-4-ene-3-one.

**Figure 5:**
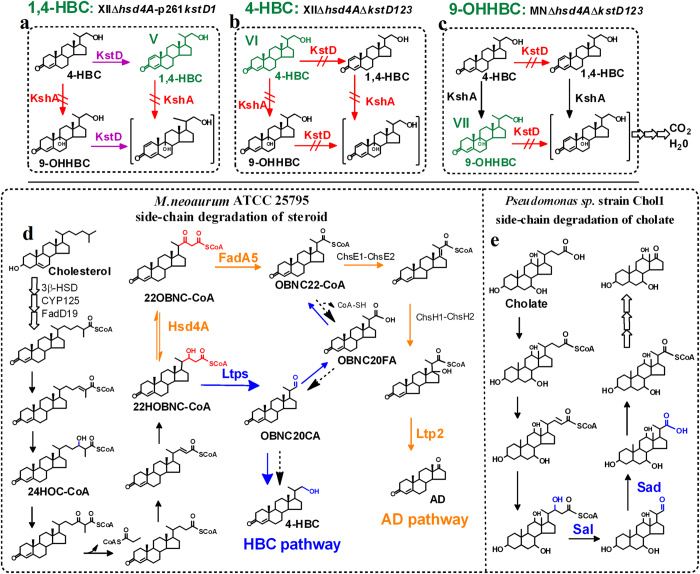
Schematic diagram of sterol catabolism and the combinatorial construction of HBCs producing strains. (**a**) The construction of strain XIIΔ*hsd4A*-p261*kstD1*. To construct XIIΔ*hsd4A*-p261*kstD1*, *hsd4A* was deleted in a *kshAs*-null mutant and then *kstD1* was overexpressed. The resulting strain XIIΔ*hsd4A*-p261*kstD1* transformed phytosterols to 1,4-HBC as the major product. (**b**) The construction of strain XIIΔ*hsd4A*Δ*kstD123*. To construct XIIΔ*hsd4A*Δ*kstD123*, *hsd4A* was deleted in a *kshAs*-null mutant and then *kstD1*, *kstD2* and *kstD3* were knocked out sequentially. The resulting strain XIIΔ*hsd4A*Δ*kstD123* transformed phytosterols to 4-HBC as the major product. (**c**) The construction of strain MNΔ*hsd4A*Δ*kstD123*. To construct MNΔ*hsd4A*Δ*kstD123*, *hsd4A* was deleted in *M. neoaurum* ATCC 25795 and then *kstD1*, *kstD2* and *kstD3* were knocked out sequentially. The resulting strain MNΔ*hsd4A*Δ*kstD123* transformed phytosterols to 9-OHHBC as the major product. 9-OHHBC, 9,22-dihydroxy-23,24-bisnorchol-4-ene-3-one. (**d**) Proposed metabolism of the sterol side chain for the production of C19 and C22 steroids in *M. neoaurum* ATCC 25795. The conversion from 22HOBNC-CoA to AD was designated as the AD pathway (orange arrows) and has been proposed as the sole pathway of sterol side-chain degradation. Here, a new pathway is proposed: the conversion from 22HOBNC-CoA to 4-HBC (designated as the HBC pathway, blue arrows). Between the two pathways, 22HOBNC-CoA is the branching-node, which leads to AD via the catalysis of Hsd4A_MN_ and leads to 4-HBC via an aldolytic reaction. The β-hydroxybutyryl-CoA moiety of 22HOBNC-CoA and acetoacetyl-CoA moiety of 22OBNC-CoA are labelled in red, which were the active substrates of Hsd4A_MN_ *in vitro*. 24HOC-CoA, 24-hydroxy-3-oxo-chol-4-en-26-oyl CoA; 22HOBNC-CoA, 22-hydroxy-3-oxo-25,26-bisnorchol-4-en-24-oyl CoA; 22OBNC-CoA, 3,22-dioxo-25,26-bisnorchol-4-ene-24-oyl CoA; OBNC22-CoA, 3-oxo-22,23-bisnorchol-4-ene-22-oyl-CoA; OBNC20FA, 3-oxo-23,24-bisnorchol-4-ene-20-formic acid; OBNC20CA, 3-oxo-23,24-bisnorchol-4-ene-20-carbaldehyde. (**e**) The proposed pathway of cholate side-chain degradation in *Pseudomonas* sp. strain Chol1^37^. *sal*, an aldol-lyase; *sad*, an aldehyde dehydrogenase. Intercepted arrows (red) indicate the blockage of the target reaction by gene deletion. Arrows in purple signify the enhanced KstD1 activity. Compounds in green are the major products accumulated in constructed strains. The compound in brackets is an unstable compound that will lead to the decomposition of steroid nucleus and further degradation. Dashed arrows indicate the proposed way for HBCs formation previously suggested by Szentirmai^32^. Arrows in arrays signify multiple reaction steps.

The catabolism of sterols is a long and complex process. Although components of the metabolic process have been identified, several key steps remain unknown, seriously limiting our understanding of sterol metabolism. *Hsd4A*_*MN*_ is a conserved gene in the gene cluster of sterol catabolism, the function of which has been speculated but not verified. This gene has been speculated as a 17β-hydroxysteroid dehydrogenase or a β-hydroxyacyl-CoA dehydrogenase in sterol metabolism^15^,^23^,^36^. In this study, Hsd4A_MN_ was first characterized as a dual-function enzyme that possesses both 17β-hydroxysteroid dehydrogenase and β-hydroxyacyl-CoA dehydrogenase activities *in vitro*. Subsequently, the dual-role of Hsd4A_MN_ in the metabolism of sterols *in vivo* was also confirmed using a *kshA*-null strain NwIB-XII as a model. The 17β-hydroxysteroid dehydrogenase activity of Hsd4A_MN_ *in vivo* was identified as the augmentation of Hsd4A_MN_ in NwIB-XII significantly decreased the production of TS and BD (Fig. 3a). However, the identification of the β-hydroxyacyl-CoA dehydrogenase activity of Hsd4A_MN_ *in vivo* was a difficult process. The deletion of *hsd4A*_*MN*_ in NwIB-XII resulted in the production of 4-HBC and 1,4-HBC, rather than TS and BD, suggesting that Hsd4A_MN_ could play another enzymatic function other than as a 17β-hydroxysteroid dehydrogenase *in vivo*. As 4-HBC and 1,4-HBC do not act as substrates for Hsd4A_MN_, these must be further derivatives of the physiological substrate of Hsd4A_MN_ during sterol side-chain degradation. Given that sterol side-chain degradation is a consensus β-oxidation, according to the characterized enzymatic properties of Hsd4A_MN_ *in vitro*, the observed β-hydroxyacyl-CoA dehydrogenase activity of Hsd4A_MN_ is likely responsible for *in vivo* sterol side-chain degradation. In the side-chain β-oxidation of sterols (Fig. 5d), there are two steryl fatty acyl-CoA esters that are considered as the substrates of β-hydroxyacyl-CoA dehydrogenase: 24-hydroxy-3-oxo-chol-4-en-26-oyl CoA (24HOC-CoA) and 22HOBNC-CoA. Compared to 24HOC-CoA, 22HOBNC-CoA seemed to be the most likely substrate of Hsd4A_MN_, as 22HOBNC-CoA could theoretically be converted to HBCs via a common retro-aldol cleavage, while the conversion of 24HOC-CoA to HBCs would be a complex and difficult biochemical process (Fig. 5d). Furthermore, the fact that Hsd4A_MN_ catalyzes the dehydrogenation of β-hydroxybutyryl-CoA *in vitro* further suggested that 22HOBNC-CoA would be a suitable substrate for Hsd4A_MN_, as β-hydroxybutyryl-CoA is an intrinsic moiety of 22HOBNC-CoA. If this was the case, 3,22-dioxo-25,26-bisnorchol-4-ene-24-oyl CoA (22OBNC-CoA) should be the direct derivative of 22HOBNC-CoA after catalysis by Hsd4A_MN_, which is a reaction known to be catalyzed by a β-hydroxyacyl-CoA dehydrogenase in the side-chain β-oxidation of sterols (Fig. 5d). Acetoacetyl-CoA and β-hydroxybutyryl-CoA are the side chain moieties of 22OBNC-CoA and 22HOBNC-CoA, respectively. As β-hydroxybutyryl-CoA and acetoacetyl-CoA could be reversibly interconverted by Hsd4A_MN_, it is conceivable that 22OBNC-CoA might also be a possible substrate of Hsd4A_MN_. 22OBNC-CoA has been characterized as a substrate of FadA5, a β-ketoacyl-CoA thiolase that catalyzes the thiolytic cleavage of 22OBNC-CoA to 3-oxo-22,23-bisnorchol-4-ene-22-oyl-CoA (OBNC22-CoA)^2^. To help decipher the above, *fadA5* was deleted in NwIB-XII (XIIΔ*fadA5*, Table S1), which resulted in a similar phenotype to XIIΔ*hsd4A*, producing 1,4-HBC and 4-HBC from the metabolism of sterols (Fig. 4a). These results demonstrated that 22OBNC-CoA similar to 22HOBNC-CoA is also a precursor of HBCs, indicating a close relationship among 22HOBNC-CoA, 22OBNC-CoA, Hsd4A_MN_ and HBCs. Structurally, the transformation of 22HOBNC-CoA or 22OBNC-CoA to HBCs requires a cleavage between the carbon_22_-carbon_23_ bond of the side chain. Biochemically, the carbon_22_-carbon_23_ bond cleavage of 22HOBNC-CoA could be performed by an aldolytic reaction, and the cleavage of 22OBNC-CoA could be achieved by a β-ketoacyl-CoA thiolysis. As the β-ketoacyl-CoA thiolysis of 22OBNC-CoA is carried out by FadA5^2^, the direct carbon_22_-carbon_23_ cleavage of 22OBNC-CoA was impossible in XIIΔ*fadA5*. However, the direct carbon_22_-carbon_23_ cleavage of 22HOBNC-CoA was feasible in XIIΔ*fadA5* as a very similar reaction has been identified in cholate side-chain degradation in *Pseudomonas* sp strain Chol1 (Fig. 5e)^37^. In contrast to 22OBNC-CoA, therefore, the conversion of 22HOBNC-CoA to HBCs was more plausible by carbon_22_-carbon_23_ cleavage. In this way, the conversion of 22OBNC-CoA to HBCs in XIIΔ*fadA5* could be reasonably attributed to the reversible conversion of 22OBNC-CoA to 22HOBNC-CoA by Hsd4A_MN_.

The conversion from 22HOBNC-CoA to AD (designated as the AD pathway, Fig. 5d) has long been proposed as the sole metabolic pathway of sterol side-chain degradation. Based on the results in this study, an additional pathway was proposed: the conversion from 22HOBNC-CoA to HBCs (designated as the HBC pathway, Fig. 5d). Between the two pathways, 22HOBNC-CoA is the branching-node, which leads to AD via the catalysis of Hsd4A_MN_ and leads to HBCs via an aldolytic reaction (Fig. 5d). Chemically, the aldolytic reaction of 22HOBNC-CoA to 3-oxo-23,24-bisnorchol-4-ene-20-carbaldehyde (OBNC20CA) could be plausibly speculated according to the similar reaction catalyzed by a *sal*-encoded aldol-lyase within the degradation of cholate side chain in *Pseudomonas* sp. strain Chol1 (Fig. 5e)^37^,^38^. Further, two aldolytic reactions have been proposed and genetically characterized in sterol side-chain degradation, including carbon17-carbon20 cleavage by aldol-lyase Ltp2^39^ and C24-branched chain cleavage by aldol-lyases Ltp3 and Ltp4^40^. For many organisms, aldehydes are a group of active compounds that are readily converted to alcohols by reductive enzymatic reactions or to carboxylic acids by oxidative enzymatic reactions. Therefore, the terminal aldehyde group of OBNC20CA has two possible further conversions. One is the oxidative transformation to 3-oxo-23,24-bisnorchol-4-ene-20-formic acid (OBNC20FA), and the other is the reductive reaction to HBCs. Obviously, the reductive conversion correlates with the results of this study, suggesting the presence of this reaction in *M. neoaurum* ATCC 25795. It must be noted that the presence of an oxidative conversion could not be determined from the results of this study. However, it is the established conversion in cholate side-chain degradation in *Pseudomonas* sp. strain Chol1 as a subsequent reaction catalyzed by an aldehyde dehydrogenase encoded by *sad* after the aldolytic reaction (Fig. 5e)^37^. Under the conditions of this study, HBCs were shown to be the dead-end products of sterols in XIIΔ*hsd4A* and XIIΔ*fadA5*, suggesting that the reductive conversion is a terminal reaction, negating further degradation of the sterol side chain. In contrast, the oxidative conversion may not affect the complete side-chain degradation, as the terminal formic acid group can be further converted to its CoA ester (i.e. OBNC22-CoA) by acyl-CoA synthetases, which will return the metabolite to the AD pathway. As the reduction and oxidation of aldehydes are common reactions in cell metabolism, the existence of the reductive conversion does not exclude the existence of the oxidative conversion in *M. neoaurum* ATCC 25795. The minor yield of C19 steroids in HBCs producing strains, such as the 1–2% molar yield of ADD in XIIΔ*hsd4A* and a small amount of AD in XIIΔ*hsd4A*Δ*kstD123*, implied that the oxidative conversion may also be active in these strains (Table 2). The molar yields of HBCs were much higher than the C19 steroids in these engineered strains; therefore, the reductive conversion was likely the dominant way to yield HBCs in *M. neoaurum* ATCC 25795, which may be closely related to the oxidation-reduction potential in mycobacterial cells.

As HBCs are valuable precursors in steroid synthesis, mycobacteria have been developed to produce HBCs^8^,^11^. Previously, 23,24-bisnorcholenic acids were considered as derivatives from the blocked AD pathway, and HBCs were speculated to be the reduced derivatives of 23,24-bisnorcholenic acids via 23,24-bisnorcholenic aldehydes (Fig. 5d)^32^. However, this study disclosed another possibility to produce HBCs in mycobacterial mutants, which has practical significance in clarifying the metabolic mechanism of C22 steroids and in the development of engineered strains for producing specific C22 steroids.

In this study, three HBCs producing strains were constructed and the productivity of each was assessed using a resting cell system that was supplemented with 40 g/l of phytosterols (Table 2). To the best of our knowledge, the production performances of these HBCs producing strains were significantly superior to other documented strains (Table 2), and the productivity of 4-HBC (0.106–0.109 g/l/h) and 1,4-HBC (0.122–0.127 g/l/h) was comparable to industrial productivity of C19 steroids (about 0.100–0.108 g/l/h), such as AD and 9-OHAD. For industrial application, the genetic and performance stability of engineered strains is highly important. In this study, gene inactivation was performed by in-frame deletion in the core region of gene, resulting in permanent inactivation of the target gene. Gene overexpression was performed using a universal mycobacterial plasmid pMV261 with kanamycin resistance. The plasmid stability of pMV261-*kstD1* in strain XIIΔ*hsd4A*-p261*kstD1* was tested under conditions lacking kanamycin addition. The results showed that the loss rate of pMV261-*kstD1* averaged less than 7% during 144 h (Fig. S6). In addition, the engineered strains developed in this study, as well as our previous study^3^,^4^, displayed stable production performances after irregular subculture for years. These results clearly demonstrated that the metabolic engineering strategies used in this study were effective in developing HBCs producing strains that were promising for industrial application, although their performances require further improvement.

## Methods

### Bacterial strains, plasmids, and chemicals

Phytosterols were purchased from Davi Biochemistry (Zhejiang, China). Hydroxypropyl-β-cyclodextrin (HP-β-CD) was purchased from Cyclochem Chemicals Co., Ltd (Kunshan, China). Cholesterol, AD, TS, ADD, BD, epi-TS, 17α-MT, acetoacetyl-CoA sodium salt, DL-β-hydroxybutyryl-CoA lithium salt, lithium acetoacetate and DL-β-hydroxybutyric acid sodium salt were obtained from Sigma-Aldrich (Shanghai, China). Other molecular biology reagents and chemicals were of the highest grade available from Sigma-Aldrich (Shanghai, China) and Thermo Scientific Fermentas (Shanghai, China).

All strains used in this study are listed in Supplementary Table S1. Oligonucleotides and plasmids are listed in Table S2. Mycobacteria were cultivated aerobically in MYC/01 medium, as described previously^3^. The stock solutions of sterols were prepared as reported^3^. *E*. *coli* DH5α used for molecular cloning and *E. coli* BL21(DE3) used for heterologous expression were grown in Luria-Bertani medium with appropriate amounts of antibiotics at 37 °C with shaking at 200 rpm.

### Gene deletion and complementation in *M. neoaurum*

The genome was isolated from *M. neoaurum* ATCC 25795 and sequenced as indicated previously^4^. Basic bioinformatics tools for sequence alignments and searching for protein homologues were performed as previously described^3^,^4^.

Unmarked in-frame gene deletion of *M. neoaurum* ATCC 25795 was based on a non-replicative suicide plasmid, p2NIL, and combined with the selectable marker cassette from pGOAL19, following a procedure described previously^3^,^41^. To delete *kshA1* and *kshA2* from *M. neoaurum*, pDEL*kshA1* and pDEL*kshA2* were constructed. *M. neoaurum* NwIB-XII was an engineered strain with the unmarked deletion of *kshA1* and *kshA2*, which served as the host to generate further gene knock-outs. The deletion of *hsd4A*_*MN*_ in *M. neoaurum* ATCC 25795 and NwIB-XII (designated as MNΔ*hsd4A* and XIIΔ*hsd4A*, respectively) was carried out using plasmid pDEL*hsd4A*. A deletion mutant of *fadA5* was constructed in NwIB-XII (XIIΔ*fadA5*) using plasmid pDEL*fadA5*. Three *kstD* genes (*kstD1*, *kstD2* and *kstD3*) were knocked out sequentially in XIIΔ*hsd4A* or MNΔ*hsd4A* using p2N-*k1*, p2N-*k2* and p2N-*k3*, respectively. Functional complementation through recombinant pMV306 (pMV306-*hsd4A*) was established as indicated^3^. A strain harboring a vacant pMV306 was used as the blank control (XIIΔ*hsd4A*-p306). All desirable mutants and complements were validated by PCR and gene sequencing. The gene sequences of *kshA1*, *kshA2*, *hsd4A*_*MN*_ and *fadA5* have been deposited in the GenBank database with the accession numbers KF573736, KF573737, KP642512 and KP642515, respectively.

### *In vivo* expression of Hsd4A_MN_ and KstD1

The coding sequences of *hsd4A*_*MN*_ and *kstD1* (GenBank ID: GQ411074.1) were amplified using PrimeSTAR® High-fidelity DNA polymerase (Takara, Dalian, China) and the primers shown in Table S2, and then were digested by *Pst*I–*Hind*III (for *hsd4A*_*MN*_) or *Bam*HI–*Hind*III (for *kstD1*) before being inserted into the corresponding sites in pMV261^42^. The resulting plasmids pMV261-*hsd4A* and pMV261-*kstD1* were electro-introduced into NwIB-XII and XIIΔ*hsd4A*, respectively. The resulting strains were then selected under 30 μg/ml kanamycin. A strain with a vacant pMV261 was used as the blank control. The plasmid stability of pMV261-*kstD1* was assesssed as indicated in Supplementary Method.

### Expression and purification of Hsd4A_MN_

The coding region of *hsd4A*_*MN*_ was ligated into vector pET-28a(+) via PCR amplification and restriction digestion (*Nco*I–*Xho*I). The resulting plasmid pET28-*hsd4A* was first replicated in *E. coli* DH5α and then transformed into *E. coli* BL21(DE3) to generate E-*hsd4A* for expression. E-*hsd4A* was cultured at 37 °C and shaken at 220 rpm in LB medium to OD_600_ 0.4–0.8 and IPTG at a concentration of 0.5 mM was added to induce Hsd4A_MN_ expression. The induced cells were further cultured for 24 h at 25 °C and shaken at 220 rpm until harvested by centrifuge at 8,000 × *g* for 20 min at 4 °C. The pellets were washed in buffer A (50 mM Tris-HCl pH7.5, 500 mM NaCl, 0.1 mM EDTA, 20 mM imidazole) plus 1 mM PMSF, centrifuged at 8,000 × *g* for 20 min at 4 °C and resuspended in 80 mL of buffer A. The cell-free extract of E-*hsd4A* was prepared by sonication for 15 min and centrifugation at 13,000 × *g* for 30 min at 4 °C. Purification of Hsd4A_MN_ was conducted using an AKTA Prime system (GE Healthcare, Shanghai, China) as indicated in Supplementary Method. The standard Bradford method, with bovine serum albumin as the standard, was applied to determine the final protein concentration^43^.

### Characterization of Hsd4A_MN_

The 17β-hydroxysteroid dehydrogenase activity of Hsd4A_MN_ was determined in triplicate as the rate of NAD^+^/NADH formation at 340 nm (ε = 6.22 mM^−1^ cm^−1^) and 30 °C, and recorded by a SpectraMax 190 (Molecular Devices; CA, USA) with the Soft-max PRO program. The assay mixture (200 μl) was comprised of 100 mM Tris-HCl (pH 7.8 for oxidation or pH 6.6 for reduction), 100 μM NAD^+^/NADH, 0.15 mM substrate and purified Hsd4A_MN_^44^,^45^. The β-hydroxyacyl-CoA dehydrogenase activity of Hsd4A_MN_ was measured by monitoring the reduction of NAD^+^ as described^46^. The assay mixture (200 μl) was comprised of 125 mM glycine-NaOH buffer (pH 9.0), 125 μM NAD^+^, 0.125 mM substrate (DL-β-hydroxybutyryl-CoA and DL-β-hydroxybutyric acid) and purified Hsd4A_MN_^46^. The acetoacetyl-CoA reductase activity of Hsd4A_MN_ was assayed by measuring the oxidation of NADH^46^. The assay mixture (200 μl) was comprised of 125 mM Tris-HCl (pH 8.0), 125 μM NADH, 0.02 mM substrate (acetoacetyl-CoA and acetoacetate) and purified Hsd4A_MN_^46^. The β-hydroxyacyl-CoA dehydrogenase and acetoacetyl-CoA reductase activities were also recorded by the SpectraMax 190 (Molecular Devices). In these enzyme reactions, one unit of specific enzyme activity was indicated as the reduction or oxidation of 1 μM NADH/NAD^+^ within 1 min per 1 mg protein at 30 °C. SigmaPlot graphical software (version 10.0, Systat Software Inc., Chicago, IL, USA) was employed to formulate the relation between [V] (reaction velocity) and [S] (substrate concentration) in terms of the Michaelis-Menten formula.

### Steroid bioconversion and analysis

In this study, two methods, including the vegetative cell biotransformation and the resting cell biotransformation, were used to perform the conversion of steroids. Vegetative cell biotransformation was carried out using MYC/02 medium with 2 g/l of cholesterol^3^. The hydroxypropyl-β-cyclodextrin resting cells were fermented with 40 g/l of phytosterols, according to the model biotransformation process^47^. The conversion was monitored for several days and sampled every 24 h. After acidification by 10% H_2_SO_4_, the samples were extracted twice using the same volume of ethyl acetate. The extracts were collected, evaporated to dryness and redissolved in methanol for analysis.

GC system 6890N (Agilent Technologies, Palo Alto, CA, USA) was used to determine cholesterol or the mixture of phytosterols^3^. High performance liquid chromatography (HPLC, Agilent Technologies) was performed on an Agilent XDB-C18 column (4.6 × 250 mm; 40 °C), as previously described^4^. Liquid chromatography-mass spectrometry (LC/MS, an AgilentTechnologies Series 1100 LC/MSD SL system with an AgilentXDB-C18, 4.6 × 250 mm column, Agilent Technologies) was used to identify the metabolites of AD, ADD, BD and TS. Electrospray ionization (ESI^+^) was carried out as follows: temperature 100 °C, desolvation temperature 300 °C, sourcecone 30 V, and desolvation 500 L nitrogen/h.

Purification of the products was performed on a preparative thin layer chromatography (HF254–366, 20 × 20 cm, Qingdao Marine Chemical Factory, China) using petroleumether/ethyl acetate (6:4; v/v) as a mobile phase. The purified products were subjected to high resolution mass spectroscopy (MS) and nuclear magnetic resonance (NMR) analyses. High resolution mass spectroscopy was performed on a Micromass GCT instrument (Micromass UK, UK) via electron ionization (EI^+^) measurements: source temperature, 250 °C; electron energy, 70 eV. The ^1^H and ^13^C NMR spectra were recorded at 300 K on a BrükerAvance 500 spectrometer (Brüker, Germany) at 125 and 500 MHz, respectively, with tetramethylsilane (TMS) as the internal standard in CDCl_3_. Chemical shifts (δ) are shown as parts per million (ppm) relative to TMS.

22-hydroxy-23,24-bisnorchol-4-en-3-one (4-HBC): ^1^H NMR: δ = 5.73 (1H, s, H-4); 3.64 (1H, dd, J_1_ = 10.3Hz, J_2_ = 2.2Hz, H-22); 3.37 (1H, dd, J_1_ = 10.2Hz, J_2_ = 7.0Hz, H-22); 1.19 (3H, s, CH_3_-19); 1.05 (3H, d, J = 6.7Hz, CH_3_-21); 0.74 (3H, s, CH_3_-18). ^13^C NMR: δ = 35.7 (C1); 34.0 (C2); 199.8 (C3); 123.8 (C4); 171.7 (C5); 32.9 (C6); 32.0 (C7); 35.6 (C8); 53.8 (C9); 38.6 (C10); 21.0 (C11); 39.5 (C12); 42.5 (C13); 55.6 (C14); 24.3 (C15); 27.7 (C16); 52.4 (C17); 12.1 (C18); 17.4 (C19); 38.7 (C20); 16.7 (C21); 67.9 (C22). MS: EI: 330.2558 [M]^+^.

22-hydroxy-23,24-bisnorchol-1,4-dien-3-one (1,4-HBC): ^1^H NMR: δ = 7.06 (1H, d, J = 10.1Hz, H-1); 6.23 (1H, d, J = 10.1Hz, H-2); 6.07 (1H, s, H-4); 3.65 (1H, dd, J_1_ = 10.3Hz, J_2_ = 2.6Hz, H-22); 3.37 (1H, dd, J_1_ = 10.3Hz, J_2_ = 7.0Hz, H-22); 1.23 (3H, s, CH_3_-19); 1.04 (3H, d, J = 5.1Hz, CH_3_-21); 0.76 (3H, s, CH_3_-18). ^13^C NMR: δ = 156.0 (C1); 127.5 (C2); 186.5 (C3); 123.8 (C4); 169.4 (C5); 33.7 (C6); 32.9 (C7); 35.6 (C8); 52.3 (C9); 43.6 (C10); 22.9 (C11); 39.3 (C12); 42.8 (C13); 55.2 (C14); 24.5 (C15); 27.7 (C16); 52.3 (C17); 12.2 (C18); 18.7 (C19); 38.7 (C20); 16.7 (C21); 67.9 (C22). MS: EI: 328.2406 [M]^+^.

9,22-dihydroxy-23,24-bisnorchol-4-en-3-one (9-OHHBC): ^1^H NMR: δ = 5.87 (1H, d, J = 1.6Hz, H-4); 3.65 (1H, dd, J_1_ = 10.5Hz, J_2_ = 3.2Hz, H-22); 3.37 (1H, dd, J_1_ = 10.5Hz, J_2_ = 7.0Hz, H-22); 1.33 (3H, s, CH_3_-19); 1.06 (3H, d, J = 6.6Hz, CH_3_-21); 0.75 (3H, s, CH_3_-18). ^13^C NMR: δ = 28.5 (C1); 34.1 (C2); 199.2 (C3); 126.8 (C4); 168.9 (C5); 31.8 (C6); 25.4 (C7); 37.4 (C8); 76.4 (C9); 44.3 (C10); 26.7 (C11); 34.9 (C12); 42.3 (C13); 49.2 (C14); 24.2 (C15); 27.6 (C16); 52.2 (C17); 11.2 (C18); 19.9 (C19); 38.7 (C20); 16.7 (C21); 67.9 (C22). MS: EI: 346.3 [M]^+^.

## Additional Information

**How to cite this article**: Xu, L.-Q. *et al.* Unraveling and engineering the production of 23,24-bisnorcholenic steroids in sterol metabolism. *Sci. Rep.* **6**, 21928; doi: 10.1038/srep21928 (2016).

## Supplementary Material

Supplementary Information

## Figures and Tables

**Table 1:** Kinetic characterization of Hsd4A_MN_ from *M. neoaurum* ATCC 25795^a^.

	Hsd4A_MN_		
Substrate^b^	*V*_max_ (μmol/min/mg)	*K*_m_(μM)	Co-factor
AD	n.d.^c^	n.d.	NADH
TS	0.169 ± 0.052	23 ± 7	NAD^+^
ADD	n.d.	n.d.	NADH
BD	0.217 ± 0.068	19 ± 4	NAD^+^
epi-TS	n.d.	n.d.	NAD^+^
17α-MT	n.d.	n.d.	NAD^+^
4-HBC	n.d.	n.d.	NAD^+^
1,4-HBC	n.d.	n.d.	NAD^+^
acetoacetate	n.d.	n.d.	NADH
DL-β-hydroxybutyric acid	n.d.	n.d.	NAD^+^
acetoacetyl-CoA	0.091 ± 0.012	101 ± 27	NADH
DL-β-hydroxybutyryl-CoA	0.113 ± 0.025	124 ± 39	NAD^+^

^a^The maximal specific activity *V*_max_ and substrate affinity constant *K*_m_ are indicated as average ± standard error (n = 3). Hsd4A_MN_ displayed distinct activities in distinct co-factor environments (NADH/NAD^+^), which is specified in the rightmost column. The activity of Hsd4A_MN_ was determined by the rate of NAD^+^/NADH formation at 340 nm (ε = 6.22 mM^−1^ cm^−1^) and 30 °C.

^b^Abbreviations: AD, androst-4-ene-3,17-dione; TS, testosterone, ADD, androst-1,4-dien-3,17-dione; BD, boldenone; epi-TS, 4-androstene-17α-ol-3-one; 17α-MT, 4-androstene-17α-methyl-17β-ol-3-one; 4-HBC, 22-hydroxy-23,24-bisnorchol-4-ene-3-one; 1,4-HBC, 22-hydroxy-23,24-bisnorchol-1,4-dien-3-one.

^c^n.d., not detected.

**Table 2:** Production performance of the constructed HBCs producing strains in this study and some previously reported HBCs producing strains^a^.

Strains	Substrates	Substrate molar conversion rate (%)	Metabolites^b^	Molar yield (%)
XIIΔ*hsd4A*	phytosterols, 40 g/l	70–73 in 144 h	**1,4-HBC**	**39**–**40**
			4-HBC	18–20
			ADD	1–2
XIIΔ*hsd4A*-p261*kstD1*	phytosterols, 40 g/l	70–73 in 144 h	**1,4-HBC**	**54**–**57**
			4-HBC	4–7
			ADD	1–2
XIIΔ*hsd4A*Δ*kstD1*	phytosterols, 40 g/l	63–66 in 144 h	**4-HBC**	**33**–**35**
			1,4-HBC	15–18
			9-OHHBC	3–4
			AD	1–2
XIIΔ*hsd4A*Δ*kstD13*	phytosterols, 40 g/l	63–66 in 144 h	**4-HBC**	**36**–**37**
			1,4-HBC	13–15
			9-OHHBC	3–5
			AD	1–2
XIIΔ*hsd4A*Δ*kstD123*	phytosterols, 40 g/l	60–62 in 144 h	**4-HBC**	**47**–**49**
			9-OHHBC	2–3
			1,4-HBC	1–2
			AD	1–2
MNΔ*hsd4A*Δ*kstD123*	phytosterols, 40 g/l	50–54 in 144 h	**9-OHHBC**	**30**–**32**
			9-OHAD	14–15
*Mycobacterium sp.* NRRL B-3683^30^	Tall oil phytosterols 1 g/l	n.m. in 192h	**1,4-HBC**	3
			**4-HBC**	Trace
			ADD	48
			AD	1
*Mycobacterium* sp. 2-4M^10^	sitosterol, 5 g/l	95–97 in 120 h	**9-OHHBC**	1.5–1.6
			**4-HBC**	n.m.^c^
			9-OHAD	48–50
			AD	21–22
			9-HCBC	0.5–0.7
			9-OHT	0.10–0.14
*M. parafortuitum complex* MCI 0617^11^	cholesterol 10 g/l	99 in 160 h	**1,4-HBC**	62
			**4-HBC**	5
			ADD	0.8
*M. neoaurum* Mut_*MN-kstD1&2&3*_^4^	phytosterols, 15 g/l	70–75 in 144 h	**4-HBC**	1–2
			9-OHAD	50–55
			AD	10–15
		

^a^The biotransformation was performed in a resting cell transformation system supplemented with 40 g/l of phytosterols for 144 h at 30 °C. Data are obtained from three independent experiments performed upon triplicate samples.

^b^Abbreviations: AD, androst-4-ene-3,17-dione; ADD, androst-1,4-dien-3,17-dione; 9-OHAD, 9α-hydroxy-androst-4-ene-3,17-dione; 4-HBC, 22-hydroxy-23,24-bisnorchol-4-ene-3-one; 1,4-HBC, 22-hydroxy-23,24-bisnorchol-1,4-dien-3-one; 9-OHHBC, 9,22-dihydroxy-23,24-bisnorchol-4-ene-3-one; 9-OHT, 9α-hydroxy-4-androsten-17β-ol-3-one; 9-HCBC, 9α-hydroxy-22-carboxy-23,24-bisnorchol-4-en-3-one. The structural information of 9-OHT and 9-HCBC is provided in Fig. S7.

^c^n.m., not mentioned.
