# Supplementary material for: Unraveling and engineering the production of 23,24-bisnorcholenic steroids in sterol metabolism
Source: Sci Rep. 2016 Feb 22;6:21928. doi: 10.1038/srep21928 (PMC4761994; doi:10.1038/srep21928)
Supplement: Supplementary Information [file srep21928-s1.pdf]

Unraveling and engineering the production of 23,24-bisnorcholenic steroids in sterol metabolism

Li-Qin Xu, Yong-Jun Liu, Kang Yao, Hao-Hao Liu, Xin-Yi Tao, Feng-Qing Wang*, Dong-Zhi Wei*

State Key Laboratory of Bioreactor Engineering, Newworld Institute of Biotechnology, East China University of Science and Technology, Shanghai 200237, China

* Corresponding author.

Feng-Qing Wang

Tel.: +86 21 64253278; fax: +86 21 64250068; e-mail address: fqwang@ecust.edu.cn (F. Q. Wang)

* Corresponding author.

Dong-Zhi Wei

E-mail address: dzhwei@ecust.edu.cn (D. Z. Wei)

Method

Plasmid stability

To test the plasmid stability of pMV261 in *M. neoaurum* ATCC 25795, strain XIIΔ*hsd4A*-p261*kstD1* was cultured in MYC/02 medium with 2 g/l of cholesterol without kanamycin addition at 30 ^{o}C. The culture was sampled at 0, 48, 96 and 144 h after incubation, and the aliquot samples were diluted appropriately and plated on LB plates supplemented with and without kanamycin. The plasmid stability was estimated in triplicate by comparing the number of colonies appeared on plates with vs. without kanamycin.

Purification of $Hsd4A_{MN}$

The purification of $Hsd4A_{MN}$ was conducted on an AKTA Prime system (GE Healthcare, Shanghai, China) equipped with a 5 ml HisTrap FF Crude column prefilled with Ni-Sepharose^{TM} 6 Fast Flow. The whole system was pre-equilibrated with 40 ml of buffer A and the cell-free extracts were loaded at a flow speed of 3–5 ml/min. Unbound fractions were washed out with buffer B (50 mM Tris-HCl pH7.5, 500 mM NaCl, 0.1 mM EDTA, 40 mM imidazole) and then the target protein was eluted using 40 ml of buffer C (50 mM Tris-HCl pH7.5, 500 mM NaCl, 0.1 mM EDTA, 200 mM imidazole). Furthermore, the eluted fractions were loaded through Q-Sepharose FF (GE Healthcare), which was pre-equilibrated with 50 mM Tris-HCl buffer (pH 7.5), and washed with the same buffer to the base line of absorption. Protein fractions were eluted from chromatography materials using 20 ml of buffer D (50 mM Tris-HCl pH7.5, 500 mM NaCl) and then concentrated to approximately 0.5 mM using Amico Ultra-4 Ultracel-30 ultrafiltration tubes (Millipore, CM, USA). Dialysis of $Hsd4A_{MN}$ was performed against 50 mM Tris-HCl (pH7.5) buffer with 0.1 mM EDTA.

Table S1

Strains used in this study.

Strains	Description	Source
Escherichia coli		
DH5α	F$^-$, φ 80d*lacZ* ΔM15,Δ(*lacZYA* -*argF*)U169, *deoR*, *recA1*, *endA1*, *hsdR17* (*rK*$^-$, *mK*$^+$), *phoA*, *supE44* , *λ*$^-$, *t hi-1*, *gyrA96*, *relA1*	Novagen
BL21(DE3)	F$^-$, *ompT, hsdSB (rB$^-$mB$^-$), gal, dcm, strain used for protein expression*	Invitrogen
E-*hsd4A*	BL21(DE3) cells carrying pET28-*hsd4A* for *in vitro*	This study
M. neoaurum		
ATCC 25795	Sterol consumer, wild-type strain[a]	[1]
NwIB-XII	Mutant strain of *M. neoaurum* ATCC 25795, with the deletion of *kshA1* and *kshA2* ,producing AD, ADD, BD and T from the conversion of sterols	This study
XIIp261*hsd4A*	Augmentation of pMV261-*hsd4A* in NwIB-XII	This study
XIIΔ*hsd4A*	Deletion mutant of *hsd4A* in NwIB-XII	This study
Cp-4A	Complementation of pMV306-*hsd4A* in XIIΔ*hsd4A*	This study
XIIΔ*hsd4A*-p261*kstD1*	Augmentation of pMV261-*kstD1* in XIIΔ*hsd4A*	This study
XIIΔ*hsd4A*Δ*kstD1*	Deletion mutant of *kstD1* in XIIΔ*hsd4A*	This study
XIIΔ*hsd4A*Δ*kstD13*	Deletion mutant of *kstD1*and *kstD3* in XIIΔ*hsd4A*	This study
XIIΔ*hsd4A*Δ*kstD123*	Deletion mutant of *kstD1*, *kstD2*, *kstD3* in XIIΔ*hsd4A*	This study
MNΔ*hsd4A*	Deletion mutant of *hsd4A* in ATCC 25795	This study
MNΔ*hsd4A*Δ*kstD1*	Deletion mutant of *kstD1*and *hsd4A* in ATCC 25795	This study
MNΔ*hsd4A* Δ*kstD13*	Deletion mutant of *kstD3* in MNΔ*hsd4A*Δ*kstD1*	This study
MNΔ*hsd4A*Δ*kstD123*	Deletion mutant of *kstD2*and *kstD3* in MNΔ*hsd4A*Δ*kstD1*	This study
XIIΔ*fadA5*	Deletion mutant of *fadA5* in NwIB-XII	This study
XIIp261	NwIB-XII harboring vacant pMV261 as a control	This study
XIIΔ*hsd4A*-p306	XIIΔ*hsd4A* harboring vacant pMV306 as a control	This study

[a] *M. neoaurum* ATCC 25795 was isolated from soil by Tsukamura and Mizuno[2] and banked in ATCC (American Type Culture Collection, accession number ATCC 25795).

Table S2

Plasmids and primers used in this study.

Name	Description
**Primers**	**forward / reverse strand**
E. coli expression	
hsd4A-f&r	TATAccatggTGAACGACAACCCGATCGACCTGT/TATActcgagAGAACCCATGAGCTCAGTTGCGGAG
M. neoaurum expression	
hsd4A-f&r	TAGCctgcagAAATGAACGACAACCCGATCGACCTGT/GCGCaagcttTCAAGAACCCATGAGCTCAGTTGCG
k1-f&r	GCAAgaattcGTGTTCTACATGACTGCCCAGGAC/ATATgtcgacGGCCTTTCCAGCGAGATGCAACGC
Deletion in mycobacteria	
Δ*kshA1*-U-F&R	ATACaagcttGGTGGGGCGCAGTACTACTCCATGT/TATAggtaccGGTGGGCCTCCCGTATCTGGTCTCT
Δ*kshA1*-D-F&R	TCTAggtaccCCACGCATGACGAGCGGGCCGGAAC/TATAgcggccgcTCTTCGCCCAACTCGACGCACAATT
Δ*kshA2*-U-F&R	TATAaagcttGACCCGGTGACGCTGCGCGACTGCG/CGCGggtaccCAGATCTGCCTAGCACGTTCGCTTC
Δ*kshA2*-D-F&R	TCCTggtaccCGGGGCATGCACCGGCCAACGGCCC/TATAgcggccgcGATCCGGTCCTTGATGGCGGTCGAG
Δ*hsd4A*-U-F&R	TATActgcagtATCGGCTGCGCCGAGACCAGTGCGA/TCGCgaattcCACGACGGCAACCTTTCCGGACAGG
Δ*hsd4A*-D-F&R	GCGCgaattcAACGGGCAGCTGTTCATCGTGTACG/CGCGaagcttTCAGGATGGTCAACCCGTTGATGAA
Δ*fadA5*-U-F&R	GCGCaagcttGTTCCTTCTTGTAGAGCTCCCACTG/TATAgaattcGTACTGGGTGACGCAGCCGCCGATG
Δ*fadA5*-D-F&R	GCGCgaattcGACATGGACAAGGTCAACGTCAACG/TATAgcggccgcGGTCGCAGATCAGGATCGGGATCTT
Q*ckd1*-U-F&R[3]	CAGTaagcttCTTCTCAGCCATACGTGGCTCCTA/TTAActgcagGTCCTGGGCAGTCATGTAGAACAC
Q*ckd1*-D-F&R[3]	TACActgcagTTGCATCTCGCTGGAAAGGCCTGA/TATAggtaccCGCGGTCAGCGTTCCGATGAACTT
Q*ckd2*-U-F&R[3]	GCTAaagcttTCCGTAATAAGAAGCCACGGAGGT/TATAgaattcGATATGGTCGACGAAGGTGCGCGA
Q*ckd2*-D-F&R[3]	TCGCgaattcGTCAAGGTAGTTCTGAGTGATCTC/TATAggatccCATACATCTCCTTGCCGTTCACAG
Q*ckd3*-U-F&R[3]	GCGCaagcttAATATCACTCGCATCAACGGATTG/TATAgaattcCGCCTTGTCGATGGTGTCATCGGT
Q*ckd3*-D-F&R[3]	TATAgaattcGAGTCACCGATGGTCCCACTGGAC/TATAggatccCCGGTTTTCGGTGTAGCTCCACAC
**Plasmids**	
pET-28a(+)	*E. coli* expression vector, Kan^R
pET28-*hsd4A*	Expression plasmid pET-28a(+) possessing orf of *hsd4A*
p2NIL	Plasmid for allelic exchange mutagenesis, non-replicative in mycobacterium[b], Kan^R

pGOAL19	Vector carrying (Hyg;Ag85p-*lacZ*;*hsp60*p-*sacB*)-PacI cassette as selection marker[4], *Amp*^{R}
pDEL*kshA1*	p2NIL-derived with selection cassette from pGOAL19 for deletion of *kshA1* in ATCC 25795
pDEL*kshA2*	p2NIL-derived with selection cassette from pGOAL19 for deletion of *kshA2* in ATCC 25795
pDEL*hsd4A*	p2NIL-derived with selection cassette from pGOAL19 for deletion of *hsd4A* in NwIB-XII
p2N-*k1*[3]	p2NIL carrying two homologous arms of *kstD1* and selection marker
p2N-*k2*[3]	p2NIL carrying two homologous arms of *kstD2* and selection marker
p2N-*k3*[3]	p2NIL carrying two homologous arms of *kstD3* and selection marker
pDEL*fadA5*	p2NIL-derived with selection cassette from pGOAL19 for deletion of *fadA5* in NwIB-XII
pMV261	Shuttle vector of *mycobacterium* and *E.coli*, carrying the heat shock (*hsp60*) promoter,*Kan*^{R}
pMV261-*hsd4A*	Recombinant pMV261, for over-expression of Hsd4A activity in mycobacteria
pMV261-*kstD1*	Recombinant pMV261, for over-expression of KstD activity in mycobacteria
pMV306	Integrative vector with single copy in *mycobacterium*, without the heat shock (*hsp60*) promoter,*Kan*^{R}
pMV306-*hsd4A*	pMV306-P*hsp60*- *hsd4A*, integrative into mycobacterial chromosomal DNA

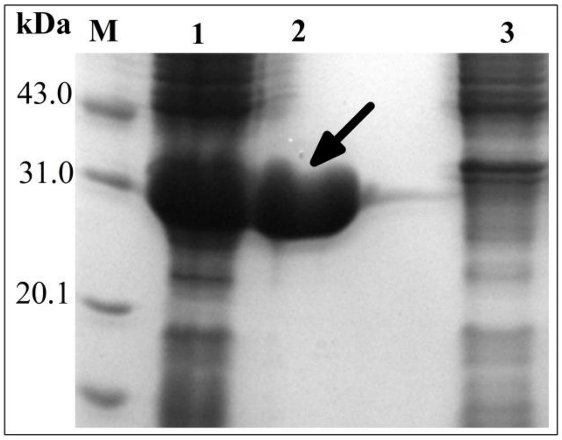

Figure S1. SDS-PAGE analysis of the expression of Hsd4A$_{MN}$. The targeted Hsd4A$_{MN}$ protein band is shown by an arrow. M, ladder of standard proteins; 1, crude extract of strain E-*hsd4A*; 2, elution fraction of purified Hsd4A$_{MN}$ with 200 mM imidazole; 3, *E. coli* BL21(DE3) harboring a vacant vector pET-28a(+).

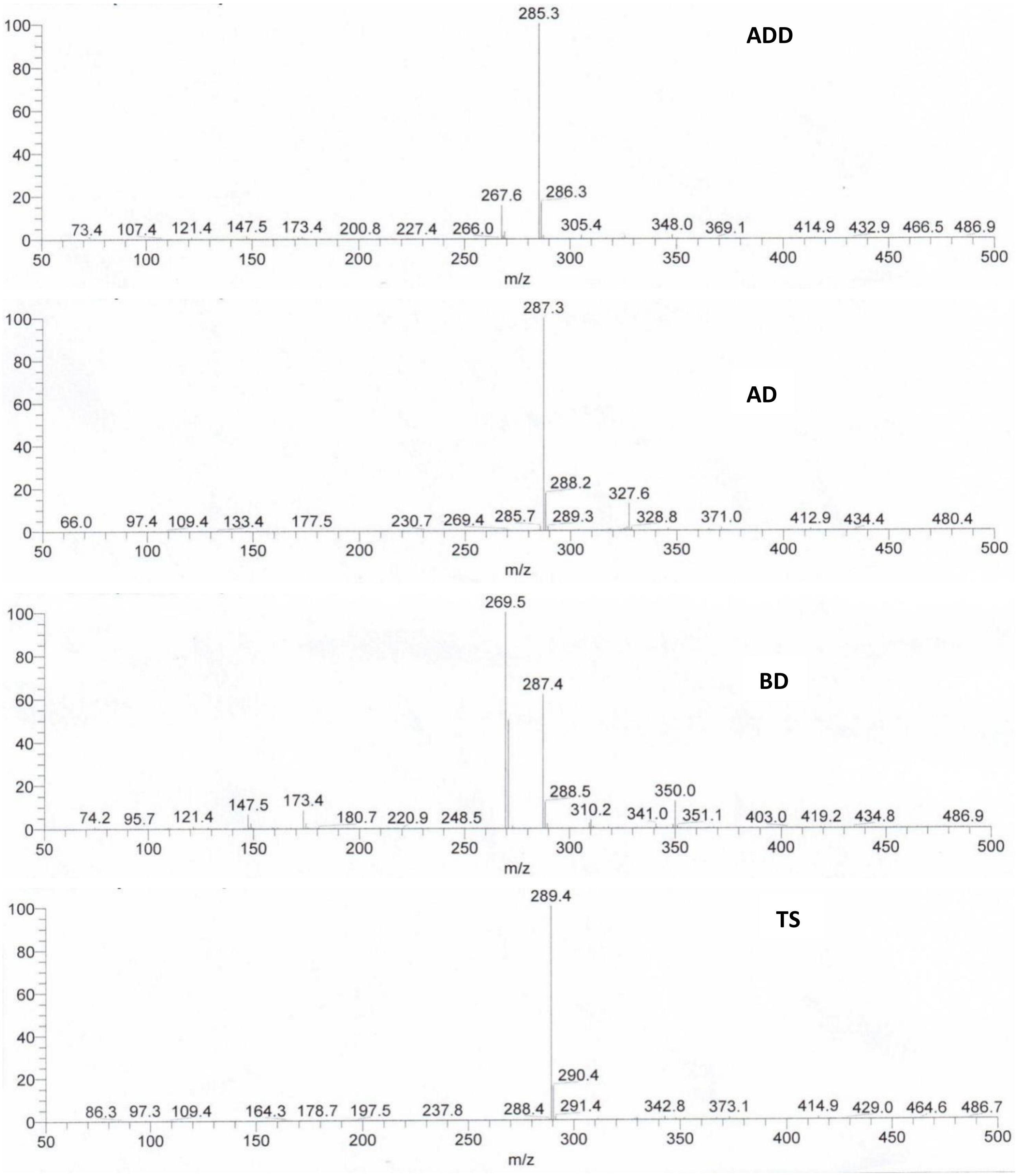

Figure S2. The mass spectrometry data of ADD, AD, BD and TS. The molecular weights of ADD, AD, BD and TS were determined as *m/z* 284.3, *m/z* 286.3, *m/z* 286.4 and *m/z* 288.4, respectively. AD, androst-4-ene-3,17-dione; ADD, androst-1,4-dien-3,17-dione; BD, boldenone; TS, testosterone.

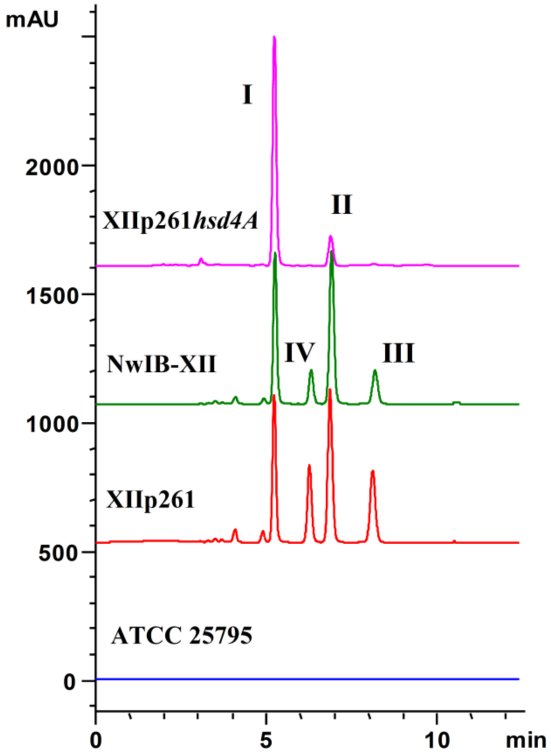

Figure S3. HPLC chromatogram comparison of the products from the transformation of 2 g/l of cholesterol in MYC/02 media at 30 °C by strains *M. neoaurum* ATCC 25795 (blue), XIIp261 (red), NwIB-XII (green) and XIIp261*hsd4A* (purple). Cholesterol can be completely degraded by *M. neoaurum* ATCC 25795 without obvious accumulation of intermediates. The catabolism of cholesterol in NwIB-XII was blocked to accumulate multiple metabolites, including ADD (I), AD (II), TS (III) and BD (IV). Strain XIIp261 transformed cholesterol to ADD, AD, TS and BD. Strain XIIp261*hsd4A* transformed cholesterol to ADD and AD. AD, androst-4-ene-3,17-dione; ADD, androst-1,4-dien-3,17-dione; BD, boldenone; TS, testosterone.

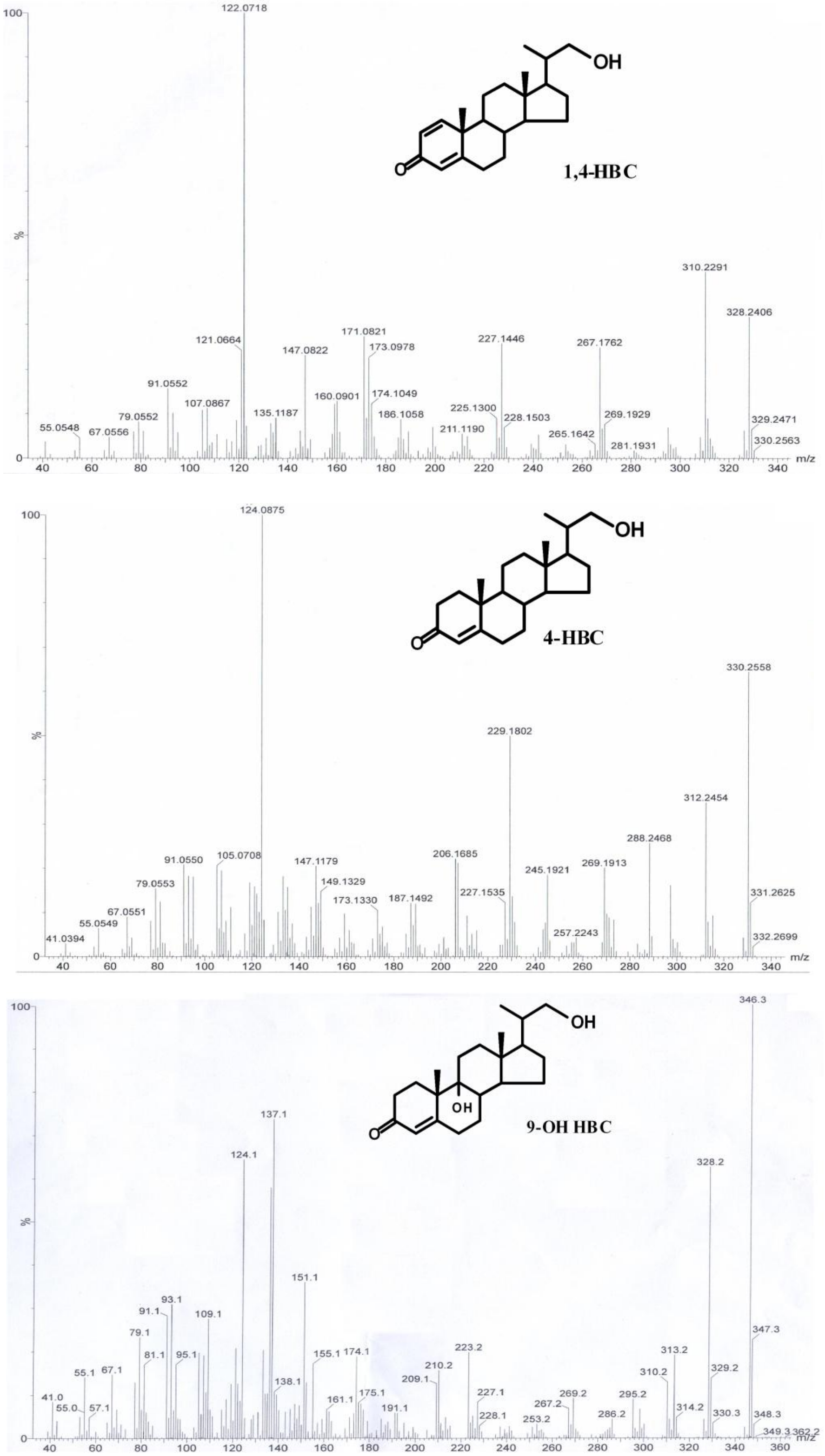

Figure S4. The mass spectrometry data of 1,4-HBC, 4-HBC and 9-OH HBC. The molecular weights of

1,4-HBC, 4-HBC and 9-OHHBC were determined as *m/z* 328.2406, *m/z* 330.2558 and *m/z* 346.3, respectively. 4-HBC, 22-hydroxy-23,24-bisnorchol-4-ene-3-one; 1,4-HBC, 22-hydroxy-23,24-bisnorchol-1,4-dien-3-one; 9-OHHBC, 9,22-dihydroxy-23,24-bisnorchol-4-ene-3-one.

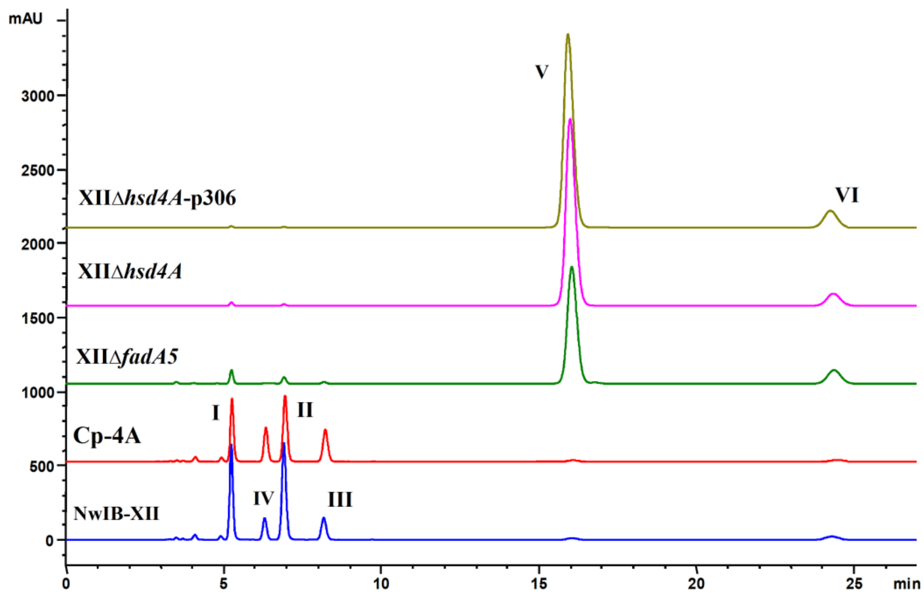

Figure S5. HPLC chromatogram comparison of the products from the transformation of 2 g/l of cholesterol in MYC/02 media at 30°C by strains NwIB-XII (blue), Cp-4A (red), XIIΔ*fadA5* (green), XIIΔ*hsd4A* (purple) and XIIΔ*hsd4A*-p306 (yellow). Both XIIΔ*hsd4A* and XIIΔ*fadA5* converted cholesterol to 1,4-HBC (V) and 4-HBC (VI). $Hsd4A_{MN}$ was complemented by integrative plasmid pMV306 in XIIΔ*hsd4A*, resulting in the Cp-4A strain, which restored the same metabolic phenotype observed in NwIB-XII. The strain XIIΔ*hsd4A*-p306 harboring a vacant pMV306 was used as the blank control of Cp-4A. 4-HBC, 22-hydroxy-23,24-bisnorchol-4-ene-3-one; 1,4-HBC, 22-hydroxy-23,24-bisnorchol-1,4-dien-3-one.

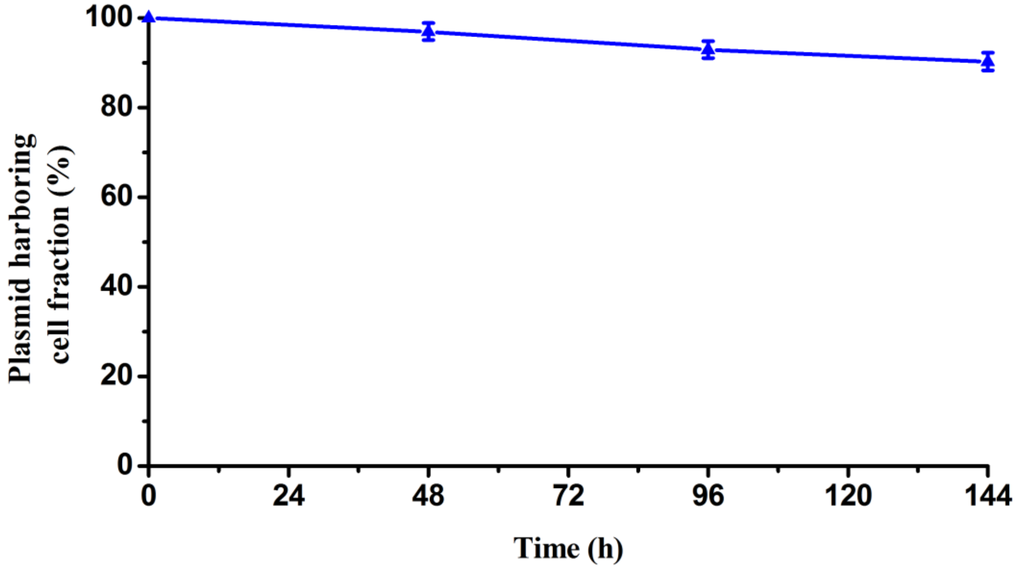

Figure S6. The plasmid stability of pMV261-*kstD1* in strain XIIΔ*hsd4A*-p261*kstD1* during 144 h in MYC/02 medium with 2 g/l of cholesterol without kanamycin addition. Triangle, the rate of cells with plasmid. Values within each curve are represented as averages ± standard deviation (error bars) from three independent determinations.

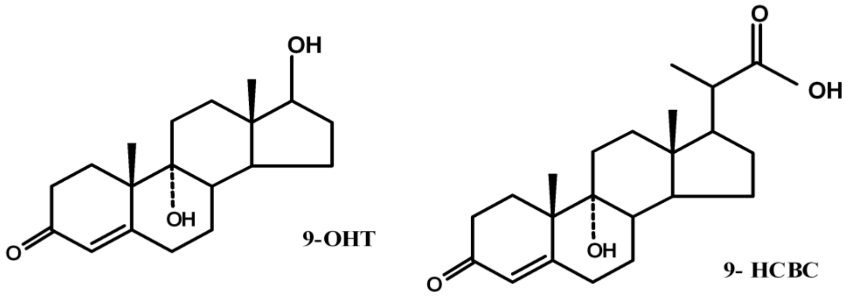

Figure S7. Structures of 9-OHT and 9-HCBC. 9-OHT, 9α-hydroxy-4-androsten-17β-ol-3-one; 9-HCBC, 9α-hydroxy-22-carboxy-23,24-bisnorchol-4-en-3-one.

References:

1. Yao, K., Wang, F.Q., Zhang, H.C. & Wei, D.Z. Identification and engineering of cholesterol oxidases involved in the initial step of sterols catabolism in *Mycobacterium neoaurum*. *Metab. Eng.* **15**, 75-87 (2013).
2. Tsukamura, M. & Mizuno, S. A new species of rapidly growing scotochromogenic mycobacteria, *Mycobacterium neoaurum*. *Med Biol (Tokyo.)* **85**, 229-233 (1972).
3. Yao, K., Xu, L.Q., Wang, F.Q. & Wei, D.Z. Characterization and engineering of 3-ketosteroid-Δ^1-dehydrogenase and 3-ketosteroid-9α-hydroxylase in *Mycobacterium neoaurum* ATCC 25795 to produce 9α-hydroxy-4-androstene-3,17-dione through the catabolism of sterols. *Metab. Eng.* **24**, 181-191 (2014).
4. Gordhan, B.G. & Parish, T. in *Mycobacterium tuberculosis protocols from methods in molecular medicine*, Vol. 54. (eds. T. Parish & N.G. Stoker) 77-92 (Humana Press Inc., Totowa, New Jersey; 2001).
